# The *in vitro* effects of black soldier fly larvae (*Hermitia illucens*) oil as a high-functional active ingredient for inhibiting hyaluronidase, anti-oxidation benefits, whitening, and UVB protection

**DOI:** 10.3389/fphar.2023.1243961

**Published:** 2023-09-20

**Authors:** Rungsinee Phongpradist, Warathit Semmarath, Kanokwan Kiattisin, Jutamas Jiaranaikulwanitch, Wantida Chaiyana, Siripat Chaichit, Yuthana Phimolsiripol, Pornngarm Dejkriengkraikul, Chadarat Ampasavate

**Affiliations:** ^1^ Department of Pharmaceutical Sciences, Faculty of Pharmacy, Chiang Mai University, Chiang Mai, Thailand; ^2^ Akkhraratchakumari Veterinary College, Walailak University, Nakhon Si Thammarat, Thailand; ^3^ Centre for One Health, Walailak University, Nakhon Si Thammarat, Thailand; ^4^ Center of Excellence in Pharmaceutical Nanotechnology, Faculty of Pharmacy, Chiang Mai University, Chiang Mai, Thailand; ^5^ Division of Product Development Technology, Faculty of Agro-Industry, Chiang Mai University, Chiang Mai, Thailand; ^6^ Center for Research and Development of Natural Products for Health, Chiang Mai University, Chiang Mai, Thailand; ^7^ Department of Biochemistry, Faculty of Medicine, Chiang Mai University, Chiang Mai, Thailand; ^8^ Anticarcinogenesis and Apoptosis Research Cluster, Faculty of Medicine, Chiang Mai University, Chiang Mai, Thailand

**Keywords:** *Hermitia illucens*, black soldier fly, oil, anti-oxidation, whitening, hyaluronidase inhibition, UV protection, cosmetic application

## Abstract

**Objective:** Larvae of *Hermitia illucens*, or black soldier fly larvae (BSFL), have been recognized for their high lipid yield with a remarkable fatty acid profile. BSFL oil (SFO) offers the added value of a low environmental footprint and a sustainable product. In this study, the characteristics and cosmetic-related activities of SFO were investigated and compared with rice bran oil, olive oil and krill oil which are commonly used in cosmetics and supplements.

**Methods:** The physicochemical characteristics were determined including acid value, saponification value, unsaponifiable matter and water content of SFO. The fatty acid composition was determined using GC-MS equipped with TR-FAME. The *in vitro* antioxidant properties were determined using DPPH, FRAP and lipid peroxidation inhibition assays. Antihyaluronidase (anti-HAase) activity was measured by detecting enzyme activity and molecular docking of candidate compounds toward the HAase enzyme. The safety assessment towards normal human cells was determined using the MTT assay and the UVB protection upon UVB-irradiated fibroblasts was determined using the DCF-DA assay. The whitening effect of SFO was determined using melanin content inhibition.

**Results:** SFO contains more than 60% polyunsaturated fatty acids followed by saturated fatty acids (up to 37%). The most abundant component found in SFO was linoleic acid (C18:2 n-6 cis). Multiple anti-oxidant mechanisms of SFO were discovered. In addition, SFO and krill oil prevented hyaluronic acid (HA) degradation via strong HAase inhibition comparable with the positive control, oleanolic acid. The molecular docking confirmed the binding interactions and molecular recognition of major free fatty acids toward HAase. Furthermore, SFO exhibited no cytotoxicity on primary human skin fibroblasts, HaCaT keratinocytes and PBMCs (IC_50_ values > 200 μg/mL). SFO possessed significant *in-situ* anti-oxidant activity in UVB-irradiated fibroblasts and the melanin inhibition activity as effective as well-known anti-pigmenting compounds (kojic acid and arbutin, *p* < 0.05).

**Conclusion:** This study provides scientific support for various aspects of SFO. SFO can be considered an alternative oil ingredient in cosmetic products with potential implications for anti-skin aging, whitening and UVB protection properties, making it a potential candidate oil in the cosmetic industry.

## Introduction

With a focus on energy conservation and sustainability, a growing demand has arisen for cost-effective sources of nutrients. The black soldier fly (*Hermitia illucens*) is a versatile insect that can thrive in various environments, particularly in hot and humid climates. Its larvae, also known as black soldier fly larvae (BSFL), are efficient decomposers of organic waste and can produce soil or frass as fertilizer and animal feed (Schmitt and de Vries, 2020). The farmed larvae, when at optimal age, can be used as an alternative source of lipid and protein in the animal nutrition industry (Driemeyer, 2016). Unlike houseflies, adult black soldier flies have no records of natural infections caused by entomopathogens in BSF (Joosten et al., 2020). Due to their unique properties and abilities, black soldier flies (BSFs) are now being used in various industries. They are considered a sustainable alternative to traditional protein sources for animal feed and an effective way to reduce food waste by converting organic waste to useful products (Kim et al., 2021). From a waste management perspective, the larvae can consume a variety of organic waste, including food scraps, fertilizer and agricultural waste and convert it to high-quality protein and oil (Wang and Shelomi, 2017). Moreover, as more people seek to ban nonbiodegradable products, BSFL can feed on organic waste and produce chitin-based bioplastics that are both biodegradable and compostable (Caligiani et al., 2018).

Insects have a significant amount of lipids in their biomass, and the highest concentration is found during the larval stage, ranging from 10% to 50% on a dry basis (da Silva Lucas et al., 2020; Franco et al., 2021). Interestingly, the oil derived from BSFL is currently being used in various cosmetic products, including soaps, lotions and shampoos (Verheyen et al., 2018; Almeida et al., 2020). The lipid composition of this oil is a combination of saturated and unsaturated fatty acids and triglycerides. Among all the lipid components, fatty acids and their derivatives are the most valuable for their beneficial properties. The larvae of BSFs are rich in fatty acids such as lauric acid, oleic acid and linoleic acid, possessing antibacterial, antifungal, and anti-inflammatory properties. They also nourish and moisturize the skin and hair, making them useful for treating various skin and scalp conditions (Xu et al., 2020; dos Santos Aguilar, 2021). Insect lipid and fatty acid profiles are influenced by several factors, including species, life stage, diet, environmental conditions, migratory flight and sex (Downer and Matthews, 1976; Franco et al., 2021). Recent studies have revealed that the composition of fatty acids in oils from BSFL is diverse. Some studies have reported that the primary fatty acids in *H. illucens* larvae are lauric acid, followed by palmitic, oleic, myristic and linoleic acids (Ushakova et al., 2016; Caligiani et al., 2019; Rabani et al., 2019). Additionally, another study has identified that the fatty acid content in the oil extracted from BSFL includes 28.8% lauric acid, 11.1% linoleic acid and 0.4% linolenic acid, which were extracted and purified from the larvae (Mai et al., 2019).

Cosmetics often incorporate fatty acids, and other components of *H. illucens* larvae oil can be applied, either isolated or as a lipidic structural complex (Almeida et al., 2020). Key components of the human epidermis, including palmitic, oleic and linoleic acid, are present in this oil (Botinestean et al., 2012). Linoleic acid plays a crucial physiologic role in maintaining the water permeability barrier of the skin (Lin et al., 2017). A deficiency in linoleic acid has been linked to dermatitis development among adults and children (Horrobin, 2000). Lauric acid is biologically active and can be transformed into monolaurin, which has antiviral, antibacterial, and antiprotozoal properties (Lieberman et al., 2006). It has also been used to treat severe antibiotic-resistant bacterial infections (Dayrit, 2015). Oleic acid activates lipid metabolism, restores the skin barrier, and helps retain moisture in the stratum corneum (Feingold and Elias, 2014). Palmitic acid and its derivatives are used as emulsifiers and emollients in creams and lotions (Mawazi et al., 2022). Fatty acids are vital components of the skin barrier and their application as formulation ingredients has increased due to their functional effects, biological activities and high biocompatibility (Duffy et al., 2017).

Several studies related to the use of BSFs in the cosmetic industry have been conducted, particularly regarding the properties and benefits of the oil produced from their larvae (Verheyen et al., 2018; Franco et al., 2021). With a lipid content ranging from 15% to 49% of total dry weight, BSFs have been found to have a high lipid yield compared with other insects (Almeida et al., 2020; dos Santos et al., 2021). Additionally, BSFs are a rich source of fatty acid derivatives that can be used as natural cosmetic ingredients, considering the high quality and quantity of fatty acids identified in their lipid composition (Müller et al., 2017; Ravi et al., 2019; Almeida et al., 2020; Franco et al., 2021). Currently, many cosmetics use palm oil, rice bran oil, olive oil, krill oil, coconut oil and lecithin as active ingredients. However, these products have become relatively expensive and palm oil in particular leaves a high environmental footprint and is non-sustainable (Huang et al., 2009). BSFs oil, on the other hand, has a fatty acid profile similar to that of palm or coconut oil, with high levels of beneficial fatty acids and anti-oxidant activity. This makes it a promising ingredient in cosmetic products (Verheyen et al., 2020). Verheyen and others have demonstrated that SFO could be a suitable alternative to palm or coconut oil to synthesize glycine-acyl surfactants, which can be used for technical applications (Verheyen et al., 2018). Overall, the potential benefits of BSFs in the cosmetic industry are significant and merit further research and exploration.

Nevertheless, information on the cosmetic-related activities of the SFO remains scarce. This study explored the potential use of BSFL in a closed farming system to generate a continuous supply of larvae, which can then be used to extract oil within 2 weeks. The resulting oil was evaluated for its potential as a new ingredient in cosmetics or functional foods, but scientific data were necessary to support its use. To this end, we conducted an evaluation of SFO as a potential oil for cosmetic application, comparing it with other commonly used oils in cosmetic production such as rice bran oil, olive oil, krill oil and lecithin. The study also discussed various aspects of the SFO preparation process, quality control, chemical composition and its cosmetic properties, compared with other oils frequently used in cosmetic applications.

## Materials and methods

### SFO oil and chemicals

SFO was supplied by Food Innovation and Packaging Center (Chiang Mai University, Chiang Mai, Thailand). Briefly, the 12-day BSFL were collected and kept frozen at −18°C before extraction. Frozen BSFL were thawed and dried in a hot air oven (FD 115, BINDER, Germany) at 80°C for 5 h and then further dried at 60°C for 3 h until moisture content achieved lower than 8%. The dried BSFL were subjected to screw pressing using a pressing machine (AS 700 W, Alangkarn Siam, Thailand) at 65–70 rpm. The obtained oil was further centrifuged using a centrifuge and filtered before filling in aluminum bags with N_2_ flushed and kept at room temperature before subjecting to further studies. The 1,1-diphenyl-2-picrylhydrazine (DPPH)*,* 2,4,6-tris (2-pyridyl)-s-triazine (TPTZ), 2,2′-azobis (2-amidinopropane) dihydrochloride (AAPH), 6-hydroxy-2,5,7,8-tetramethylchromane-2-carboxylic acid (Trolox), and linoleic acid were purchased from Sigma Chemical Co. (St. Louis, MO, United States). Rice bran oil (5,000 ppm oryzanol, King, Thailand) and olive oil (Bertolli, extra virgin, Italy) were purchased from a local gourmet store. Krill oil and lecithin (food grade) were purchased from Xi’an Natural Field Bio-Technique, China. Methanol, dichloromethane, ethanol, acetonitrile and other solvents were purchased from RCI Labscan, Bangkok, Thailand. Dulbecco’s modified Eagle medium (DMEM), trypsin, and penicillin-streptomycin were supplied by Gibco (Grand Island, NY, United States). Fetal bovine serum (FBS) was supplied by GE Healthcare (Little Chalfont, United Kingdom). The 3-(4,5-dimethylthiazol-2-yl)-2,5-diphenyltetrazolium bromide (MTT) dye, 2′,7′-dichlorofluorescin diacetate (DCF-DA), kojic acid, and arbutin were purchased from Sigma-Aldrich (St. Louis, MO, United States).

### Physicochemical characterization of SFO

The pH value of SFO was determined at 25°C using a pH meter (Metrohm, Herisau, Switzerland), which was calibrated using pH 4 and 7 buffers. Viscosity of SFO was measured at 25°C with a Brookfield R/S-CC rheometer using a Bob and Cup format (Brookfield, MA, United States). The measuring system was the CC48 DIN with a controlled shear rate. The values of acids, saponification and unsaponification were examined according to the method of <401> USP 40 (Pharmacopeia, 2017). The water content was determined by the well-established methods of Karl Fischer titration using Karl Fischer V20 Titrator (Mettler Toledo, OH, United States). All measurements were carried out in triplicate.

### Fatty acid composition analysis

Lipids were extracted using a modified version of the method described by Lepage and Roy, (1986). Three grams of sample were homogenized for 4 min × 15 min with 12 mL of dichloromethane: methanol (2:1 v/v). The sample was filtered, and potassium chloride was added. A biphasic separation was obtained by centrifugation (2000 rpm, 10 min). After that, the supernatant was collected and methanolic solution of 0.5 mM NaOH was then added for incubating at 100°C, 15 min. Methylation was catalyzed by 2 mL 14% BF_3_-methanol complex in methanol. To extract the fatty acid methyl esters, 5 mL 20% NaCl and 0.5 mL hexane were added, followed by centrifugation for 5 min at 1,000 rpm. The upper phase (containing the fatty acid methyl esters; FAME) was collected for the analysis for fatty acid profile using GC-MS (Trace GCultra, Italy/Polaris Q, United States), equipped with a TR-FAME (60 m × 0.25 mm, film thickness 0.25 µm). The carrier gas was helium, and the detector temperature was 230°C with a split ratio (20:1), and 1 µL was injected (split ratio 1:20). Fatty acid composition was calculated using the fatty acid methyl ester internal standard and expressed as percentage of total detected fatty acids. All measurements were performed by the Research and Service Laboratory, The Halal Science Center, Chulalongkorn University, Bangkok, Thailand under the Halal GMP/HACCP and Halal-QHS/ISO 22000.

### Determination of anti-oxidant activities

Anti-oxidation potential of SFO was determined in comparison with other oils and a positive control, Trolox (5 mM), using different methods including DPPH, FRAP and lipid peroxidation inhibition assays. The free radical scavenging property of samples was evaluated using the DPPH assay (Kiattisin et al., 2019). DPPH solution at a concentration of 167 µM was prepared in absolute ethanol. In brief, DPPH solution was added in a 96-well plate and mixed with an oil sample (20 μL), mixed well. Following that, the sample was incubated in the dark at 25°C for 30 min. The absorbance was then measured at 520 nm using a microplate reader (SPECTROstar Nano, Offenburg, Germany). The percentage of inhibition was determined using the equation below.

$$\%\;Inhibition=\left[\frac{C-S}{C}\right]x\;100$$

where *C* is the absorbance of control, and *S* is the absorbance of sample. The experiments were performed in triplicate.

The reducing capacity of samples was investigated using the ferric reducing anti-oxidant power (FRAP) assay (Kiattisin et al., 2019). The FRAP reagent was prepared by mixing 300 mM acetate buffer, 20 mM ferric chloride solution and 10 mM TPTZ. Then FRAP reagent was mixed with each undiluted sample in a 96-well plate and incubated at room temperature for 5 min. The sample was then measured for absorbance at 595 nm using a microplate reader (SPECTROstar Nano, Germany). The experiments were performed in triplicate, and the results were expressed as a FRAP value or ferrous sulfate (FeSO_4_) equivalent per Gram of sample.

The lipid peroxidation inhibition property of samples was determined using the linoleic acid thiocyanate method (Poomanee et al., 2021). Each sample was mixed with linoleic acid in methanol, phosphate buffer pH 7.0, DI water and AAPH in a test tube. The sample was then incubated in a water bath at 45°C for 4 h. After, the reaction mixtures were mixed with 75%v/v methanol, 10% ammonium thiocyanate solution and 2 mM ferrous chloride in a 96-well plate. After precisely 3 min, the absorbance was measured at 500 nm using a microplate reader (SPECTROstar Nano, Germany). The experiments were performed in triplicate. The percentage of lipid peroxidation inhibition was determined using the equation below.

$$\%\;Inhibition=\left[\frac{C-S}{C}\right]x\;100$$

where *C* is the absorbance of the control, and *S* is the absorbance of the sample.

### Determination of hyaluronidase (HAase) inhibitory activity

The inhibition of HAase was determined by measuring *N*-acetyl-glucosamine level, which was produced by the interaction of sodium hyaluronate with HAase (Laothaweerungsawat et al., 2020). Before each experiment, the HAase enzyme activity was measured, and only enzyme activity more than 90% was employed. In brief, the oil samples were incubated for 10 min with 15 unit/mL HAase at 37°C. Following that, 0.03% w/v hyaluronic acid (HA) in phosphate buffer pH 5.35 was added and incubated for 45 min at 37°C. HA was then precipitated using acid bovine serum albumin, produced from sodium acetate, acetic acid and bovine serum albumin. Using a multimode detector, the absorbance of the resultant combination was measured at 600 nm (BMG Labtech, Offenburg, Germany). Every experiment was carried out in triplicate. The HAase inhibition was determined using the equation below.

$$\%\;Inhibition=\left[1-\left(\frac{a}{b}\right)\right]x\;100$$

where *a* is the absorbance of the mixtures comprising oil sample, HAase, HA and bovine serum albumin solution, and *b* is the absorbance of the mixtures comprising HAase, HA and bovine serum albumin solution without oil samples. As a positive control, oleanolic acid, an anti-inflammatory and anti-oxidative triterpenoid compound was employed.

### Molecular docking of promising compounds toward the HAase enzyme

In this study, a crystal structure of human HAase was retrieved from Protein Data Bank (PDB ID: 2PE4) (Chao et al., 2007). All the hetero-atoms and water molecules were removed from the protein structure. The 3D structures of free fatty acids and oleanolic acid were obtained in the PubChem database (https://pubchem.ncbi.nlm.nih.gov/) and further geometry optimized using Gaussian09w with the B3LYP/6-311G (d,p) basis set (Hehre et al., 1970). The hydrogen atoms and Gasteiger’s charges were assigned using AutoDockTools 1.5.6 (Morris et al., 2009) The docking simulation was performed using AutoDock Vina (Trott and Olson, 2010). A grid box (25 × 25 × 25 Å^3^) was created from the center of the catalytic site (X: 41.945, Y: -21.787 and Z: -16.336). The structures with the lowest binding energies were selected for analysis, and the molecular recognition was identified using LigandScout 4.4.8 (Wolber and Langer, 2005). Structural figures were generated using PyMOL (PyMOL Molecular Graphics System, Version 2.0 Schrödinger, LLC).

### Cell culture

Primary human skin fibroblasts were aseptically isolated from an abdominal scar after a surgical procedure involving a cesarean delivery at the surgical ward of CM Maharaj Hospital, Chiang Mai University, Chiang Mai, Thailand (Study code: BIO-2558-03541 approved by Medical Research Ethics Committee, Chiang Mai University). The protocol of isolating and preparing the primary fibroblasts were followed according to a described protocol (Limtrakul et al., 2016).

HaCaT cells (human keratinocytes) and B16-F10 melanoma cells were purchased from ATCC (VA, United States). All cell types were cultured in DMEM with 10% FBS and supplemented with 100 U/mL penicillin and 100 µg/mL streptomycin. The cells were maintained in a 5% CO_2_ humidified incubator at 37°C.

Human blood samples were obtained from the Blood Bank Laboratory, Maharaj Hospital, Chiang Mai, Thailand which remained in the laboratory and could not be identified by hosts. Human Peripheral Blood Mononuclear Cells (PBMC) were prepared using Ficoll-hypaque density gradient centrifugation. Cells were rested in RPMI 1640 medium supplemented with 10% fetal bovine serum, 2 mM L-glutamine, 100 IU/mL penicillin and 100 µg/mL streptomycin, overnight before the experiment.

### Cytotoxicity effects using the MTT assay

The effects of SFO on cell viability were determined using the MTT assay as described. Briefly, the cells were treated with increasing concentrations of SFO (0–200 μg/mL) in culture medium or culture medium alone (vehicle control) for 24 and 48 h. Following the SFO treatment, the cells were incubated with 10 μL of 0.5 mg/mL MTT in PBS for 4 h. The culture supernatant was then removed, and the culture was re-suspended with 200 μL of DMSO to dissolve the MTT formazan crystals. The absorbance was measured at 540 and 630 nm using a UV-visible spectrophotometer, and the assay was performed in triplicate.

### Determination of *in situ* intracellular ROS inhibitory effects

Intracellular ROS after UVB irradiation was determined using a DCF-DA assay, as described (Eruslanov and Kusmartsev, 2010; He et al., 2017). The fibroblasts (8.0 × 10^5^ cells/well) were seeded in a 96-well plate for 24 h. After that, the cells were exposed to UVB (15 mJ/cm^2^) using a ultraviolet crosslinker machine. The cells were then treated with or without SFO (0–200 μg/mL) for 24 h. After that, the cells were washed with PBS, 10 µM of DCF-DA was added and the cells were incubated for a further 30 min. The fluorescent intensity was measured at an excitation wavelength of 485 nm and an emission wavelength of 525 nm. The inhibitory effects of SFO on intracellular ROS production at 24-h post UVB exposure was calculated and compared with the control of UVB-irradiated fibroblast cells.

### Determination of melanin content inhibition

B16-F10 cells (1 × 10^5^ cells/dish) were seeded in a 60 × 15 mm^2^ dish and incubated for 24 h at 37°C, 5% CO_2_. After that, the cells were treated with 50 μg/mL of kojic acid or arbutin (positive controls) or SFO, olive oil and linoleic acid at the concentrations of 50 µg/mL. The cells were incubated for 48 h and then collected and washed with PBS. NaOH was added and the cells were incubated at 80°C for 30 min. The absorbance was determined using a microplate reader at 405 nm.

### Statistical analysis

Data were collected from three independent experiments and are shown as mean ± SD. Statistical analysis was performed using t-tests, ANOVA and Tukey’s multiple comparison test, using SPSS, Version 19.0 (IBM^®^ SPSS Statistics, Armonk, NY, United States). A *p-value* less than 0.05 was considered statistically significant.

## Results

### Physicochemical characterization and fatty acid composition analysis of SFO

The oil obtained from SFO was subjected to physicochemical and fatty acid composition analysis to validate its quality. SFO was found to possess a brownish color and oily texture. Table 1 summarizes the physicochemical characteristics of SFO, including pH (6.0), viscosity (52 cPa.s), unsaponifiable matter (0.72 + 0.17%), acid value (3.00 ± 0.81 mg KOH/g) and water content (0.13%w/w).

**TABLE 1:** Chemical properties of SFO.

Chemical property	Result
pH	6.00 ± 0.15
Viscosity (cPa.s)	52.0 ± 0.3
Unsaponifiable matter (%)	0.72 + 0.17
Acid value (mg KOH/g)	3.00 ± 0.81
Saponification value (mg KOH/g)	197.1 ± 9.5
Water content (%w/w)	0.13 ± 0.02

**FIGURE 1:**
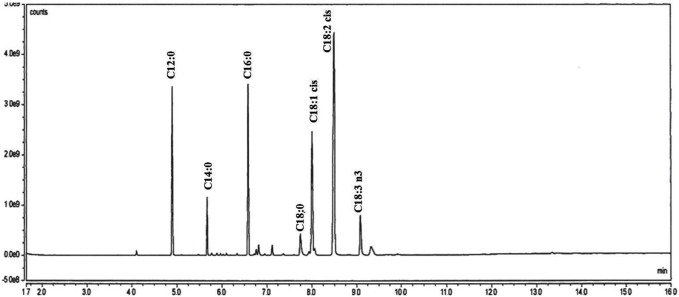
GC/MS analysis of fatty acids in black soldier fly oil.

In addition to the physiochemical evaluation of SFO, the fatty acid composition of SFO was analyzed using GC-MS. The lipid extracts of SFO were displayed as a chromatogram, and the percentage of fatty acid composition was calculated and is presented in Figure 1 and Table 2. Linoleic acid was found to be the predominant fatty acid, with a content of 34.19%, followed by oleic acid (15.95%), palmitic acid (15.93%) and lauric acid (13.25%). The remaining fatty acids, including capric acid, myristic acid, palmitoleic acid, margaric acid, steric acid and linolenic acid, were all found present in amounts less than 10%. SFO contained high percentages of polyunsaturated fatty acids (PUFA) of up to 60%, followed by saturated fatty acids of up to 37%.

**TABLE 2:** Fatty acid profile of black soldier fly oil.

Fatty acid	Percentage (%)
Capric acid (C10:0)	0.33 ± 0.02
Lauric acid (C12:0)	13.25 ± 1.81
Myristic acid (C14:0)	3.82 ± 0.19
Palmitic acid (C16:0)	15.93 ± 0.56
Palmitoleic acid (C16:1)	0.95 ± 0.16
Margaric acid (17:0)	0.99 ± 0.03
Steric acid (C18:0)	3.43 ± 0.32
Oleic acid (C18:1 n-9 cis)	15.95 ± 0.64
Linoleic acid (C18:2 n-6 cis)	34.19 ± 0.44
Linolenic acid (C18:3 n-3)	5.68 ± 0.33

### Determination of anti-oxidant activities of SFO

The use of natural oils in pharmaceutical and cosmetic products for their anti-oxidant properties has been well established. Therefore, our study aimed to examine the anti-oxidant activities of SFO compared with Trolox and commonly used oils in the cosmetic and nutraceutical industries. We evaluated the anti-oxidant activity of SFO using different assays including DPPH, radical scavenging, FRAP, and lipid peroxidation inhibition tests, and compared it with rice bran oil, olive oil, krill oil, lecithin and linoleic acid.

**FIGURE 2:**
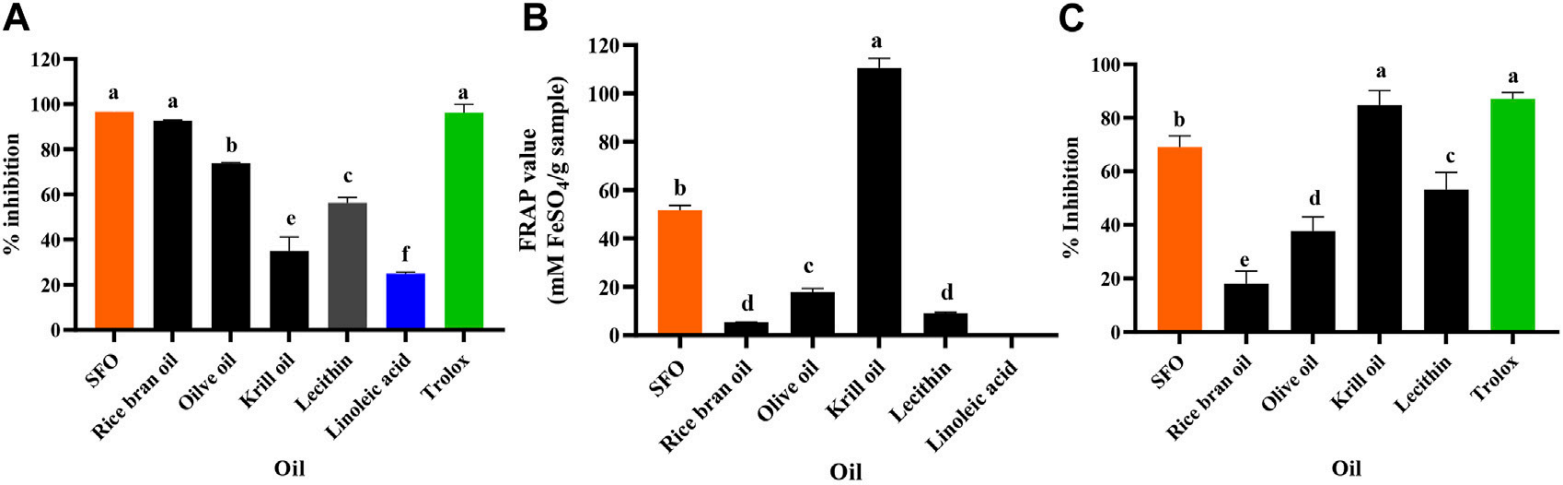
Anti-oxidant activity evaluated with **(A)** DPPH, **(B)** FRAP and **(C)** Lipid peroxidation inhibition assays of SFO, rice bran oil, olive oil, lecithin, linoleic acid, and Trolox (a, b, c, d, e and f denote significant differences among samples at *p* < 0.05).

The results of the anti-oxidant activity tests of SFO, rice bran oil, olive oil, krill oil, lecithin and linoleic acid are shown in Figures 2A–C. According to the results, SFO exhibited multiple mechanisms to interrupt the oxidation processes compared with the other oils. The DPPH assay is a well-known method for assessing stable free radical scavenging activity (Rahman et al., 2015). Neat SFO showed the highest DPPH radical scavenging activity (96.61% inhibition) which was significantly higher than that of the other oils and even linoleic acid (24.93%–92.69%, *p* < 0.05), except for rice bran oil, which was higher but did not differ significantly (Figure 2A). The free radical scavenging activity of SFO is comparable to the activity of 5 mM Trolox, a positive control.

The FRAP assay directly measures anti-oxidants (or reductants) in a sample and represents the corresponding concentration of electron-donating anti-oxidants with reduced ferric iron (Fe^3+^) compared with the ferrous ion (Fe^2+^) (Kiattisin et al., 2019). In our study, the FRAP assay showed that krill oil exhibited the highest reducing power (110.59 ± 3.99 mM FeSO_4_/g sample), followed by SFO (51.61 ± 2.08 mM FeSO_4_/g sample), while the common oils exhibited significantly lower reducing power. Interestingly, the positive control, Trolox (5 mM) exhibited significantly higher FRAP value of 28,724 mM FeSO_4_/g sample that is significantly high and could not be plotted with the oils’ FRAP values. Contrastingly, linoleic acid was inactive, implying that it could not be responsible for the reducing ability of SFO (Figure 2B). Lipid peroxidation in cells leads to degrading the lipid bilayer composing cell membranes, and the end-products can further promote mutagenesis or protein oxidation, disturbing cellular homeostasis (Ali et al., 2022). Inhibition of chain reactions of lipid peroxidation is promoted in anti-aging discovery. Similar to the FRAP assay, the lipid peroxidation inhibition assay showed that SFO (69.03%) had significantly higher activity than that of the other oils (18.05%–53.17%) except for krill oil and the positive control, Trolox (5 mM), exhibiting the highest lipid peroxidation inhibition activity (84.71% and 87.12%, respectively) (Figure 2C).

### HAase inhibitory activity of SFO and molecular docking of its major fatty acids toward the HA enzyme

**FIGURE 3:**
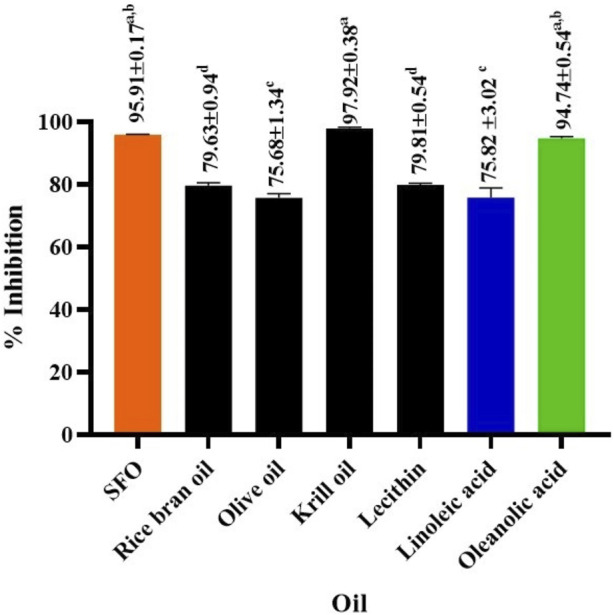
Effect of SFO, rice bran oil, olive oil, krill oil, lecithin, linoleic acid and oleanolic acid (a positive control) on hyaluronidase inhibitory activity., (a, b, c and d denote significant differences among samples at *p* < 0.05).

The study investigated whether SFO and its potential fatty acids could inhibit the activity of HAase, which primarily degrades HA in the skin. The disappearance of HA in the skin can result in significant histochemical changes associated with aging (Farage et al., 2008). In this study, we investigated whether SFO and its potential fatty acid could possess inhibitory effects on HAase activity. The HAase inhibitory activity of SFO, rice bran oil, olive oil, krill oil, lecithin, linoleic acid as well as the positive control, oleanolic acid, are shown as the %inhibition in Figure 3. SFO (95.91%) and krill oil (97.92%) exhibited strong HAase inhibition comparable with the positive control, oleanolic acid (94.74%). Linoleic acid, a major fatty acid in SFO, also showed HAase inhibitory activity, but to a lesser extent than the crude oil (75.82%).

**FIGURE 4:**
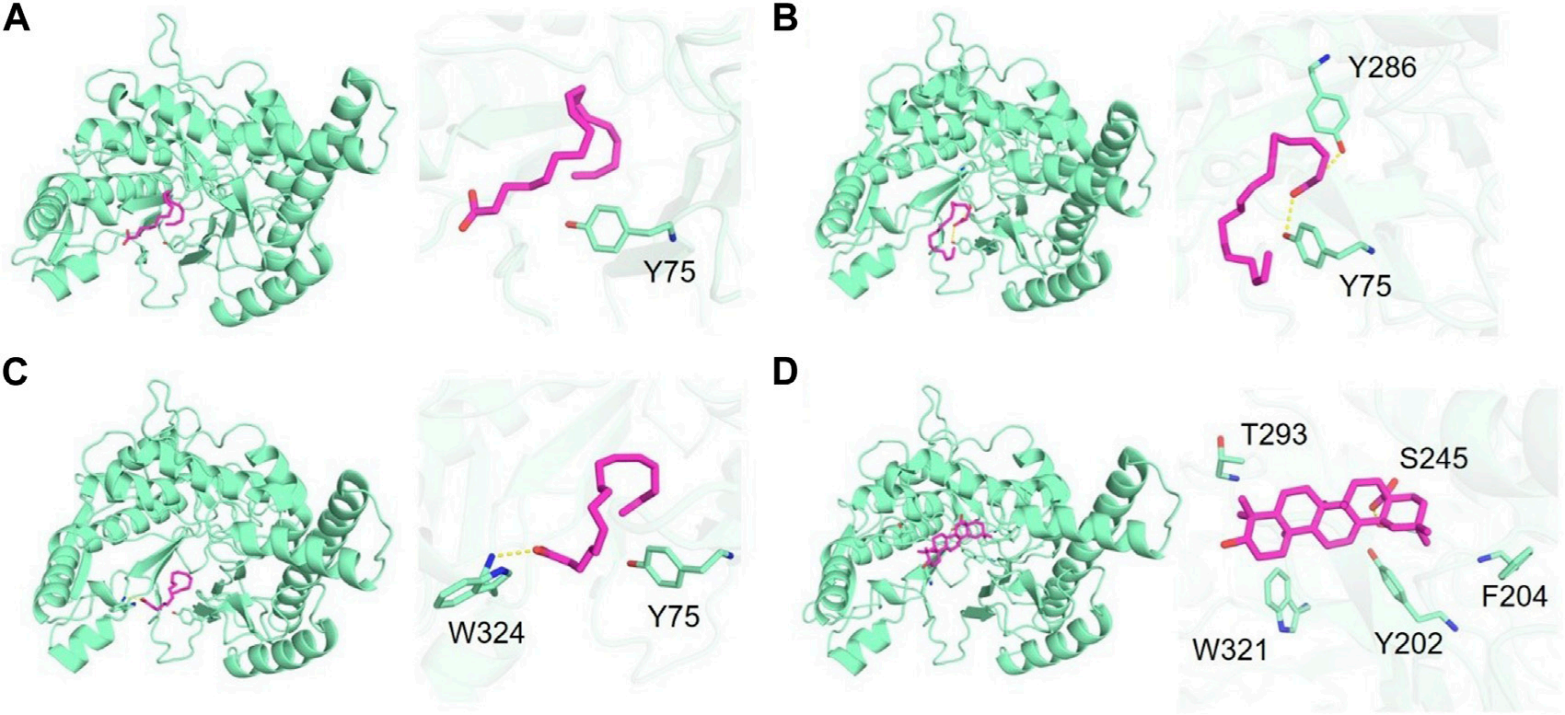
Binding of hyaluronidase (green) interacting with free fatty acids (magenta) including **(A)** linoleic acid, **(B)** oleic acid, **(C)** palmitic acid and **(D)** positive control (oleanolic acid).

To further understand the mechanism of enzyme inhibition, molecular docking studies were conducted on major fatty acid components of SFO including linoleic acid, oleic acid and palmitic acid, as well as the positive control, oleanolic acid. Molecular docking was carried out to investigate the binding interactions and molecular recognition of selected free fatty acids toward HAase (PDB: 2PE4). The molecular interactions of the docked complexes are depicted in Figures 4A–D. Linoleic acid, oleic acid and palmitic acid were positioned at the binding pocket of the HAase enzyme. The fatty acid-binding pocket was mostly hydrophobic with residues including M71, I73 and Y75, where the tail of fatty acids fits well (Figures 4A–C). The positive compound was well accommodated within the active site of the enzyme by forming a hydrogen bond with S245 and hydrophobic interaction with residues Y202, F204, T293 and W321 (Figure 4D). The docking results showed that linoleic acid exhibited the highest binding affinity to HAase at −6.5 kcal/mol, followed by oleic acid and palmitic acid with binding energies of −6.0 and −5.8 kcal/mol, respectively. The positive control, oleanolic acid, showed a strong binding score of −8.5 kcal/mol, which was consistent with the *in vitro* experiment.

### Effects of SFO on cells viability

**FIGURE 5:**
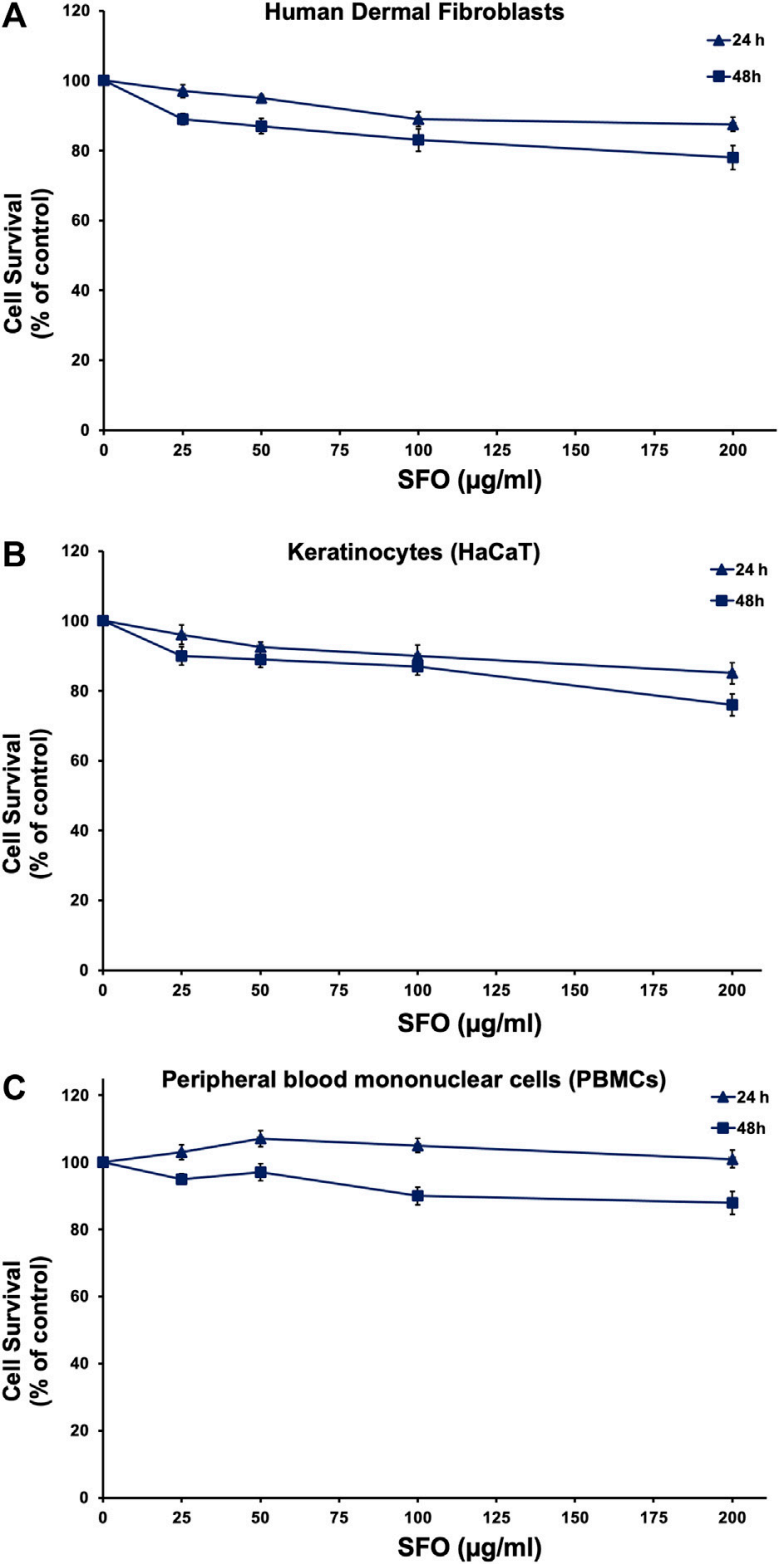
Effect of SFO on human normal cells viability, Human dermal fibroblasts **(A)**, HaCaT human keratinocytes **(B)** and human PBMCs **(C)** were treated with varying concentrations of SFO for 24 h and 48 h. The viability of cells was assessed using the MTT assay, and the results are presented as mean ± S.D. of three independent experiments.

To examine the potential use of SFO in cosmetic preparation, we conducted a safety assessment by determining its cytotoxicity effects on human skin cells (keratinocytes and fibroblasts) as well as human white blood cells (PBMC), using the MTT assay. Primary human skin fibroblasts, HaCaT keratinocyte cell line and PBMCs were treated with increasing concentrations of SFO for 24 and 48 h. Our results, as shown in Figures 5A–C, indicated that even at a high concentration of 200 μg/mL, SFO did not display cytotoxicity effects on human dermal fibroblasts (Figure 5A), HaCaT keratinocytes (Figure 5B) or PBMCs (Figure 5C) after 24 and 48 h of incubation. The IC_50_ value for all types of cells was greater than 200 µg/mL of SFO. Therefore, our findings suggested that SFO is safe for use in cosmetic applications as it did not show any cytotoxicity effects on human normal cells.

### *In-situ* ROS scavenging effects of SFO on UVB-irradiated human dermal fibroblasts

**FIGURE 6:**
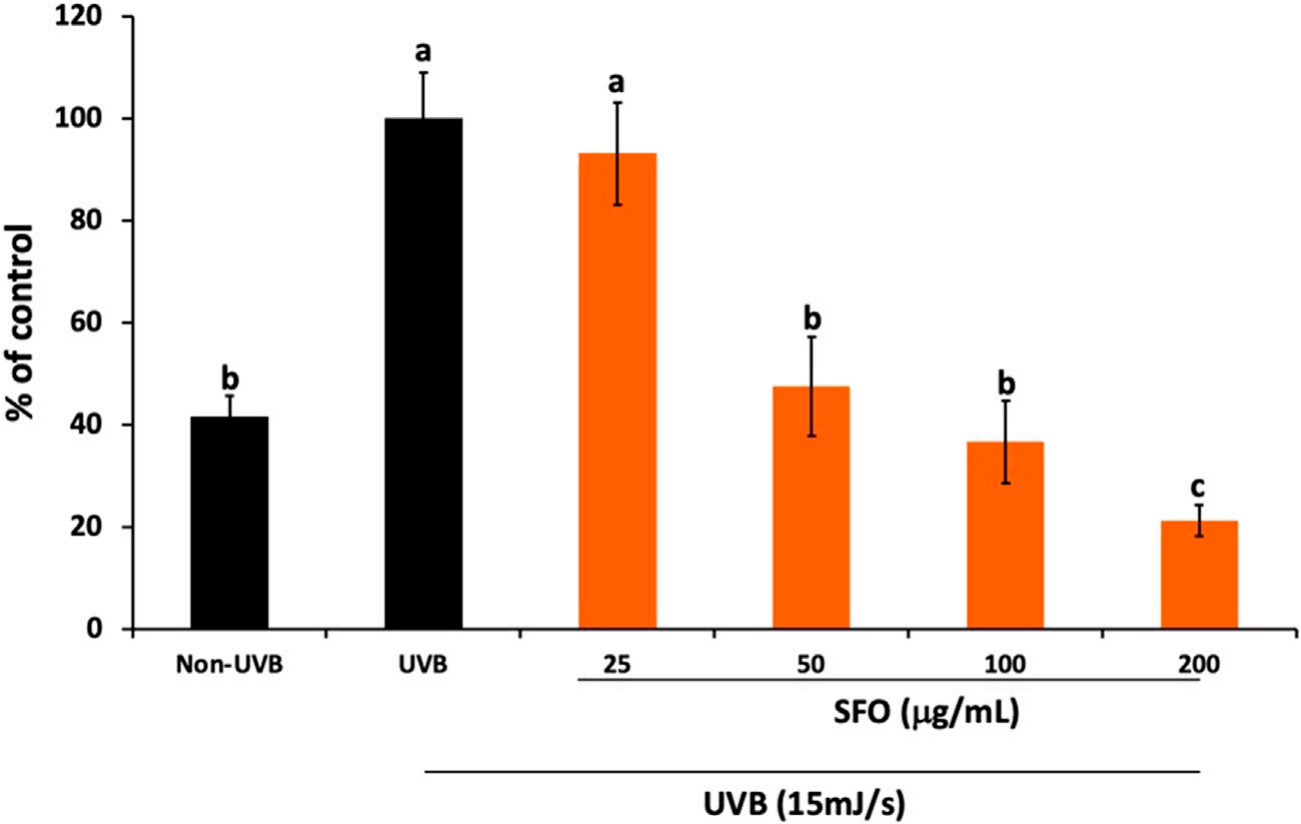
*In-situ* anti-oxidant activity of SFO. The inhibition of intracellular ROS upon UVB irradiation in human dermal fibroblasts was determined using the DCF-DA fluorescent assay. The data is presented as mean ± S.D. of 3 independent experiments (a, b and c denote significant differences among samples at *p* <0.05).

We initially evaluated the *in vitro* anti-oxidant properties of SFO and found it exhibited strong anti-oxidant effects. To further confirm its anti-oxidant capacity *in-situ*, we conducted DCF-DA assays to determine the intracellular ROS scavenging properties in UVB-irradiated dermal fibroblasts. The results are shown in Figure 6. The findings indicated that UVB radiation increased ROS generation in human dermal fibroblasts by approximately 60% when compared with nonirradiated fibroblasts. Interestingly, when the cells were pretreated with SFO, increasing concentrations of SFO significantly reduced intracellular ROS production in UVB-irradiated fibroblasts in a dose-dependent manner at post-24 h of UVB exposure (*p* < 0.05). At this exposure time, SFO at the concentration of 200 µg/mL inhibited the intracellular ROS production to less than 20% when compared with the irradiated fibroblasts group. These findings demonstrated that SFO has the potential capacity to reduce the generation of ROS through its ROS scavenging ability. Thus, SFO exhibited antioxidant properties in both *in vitro* and *in situ*.

### The effects of SFO on the inhibition of melanin content

**FIGURE 7:**
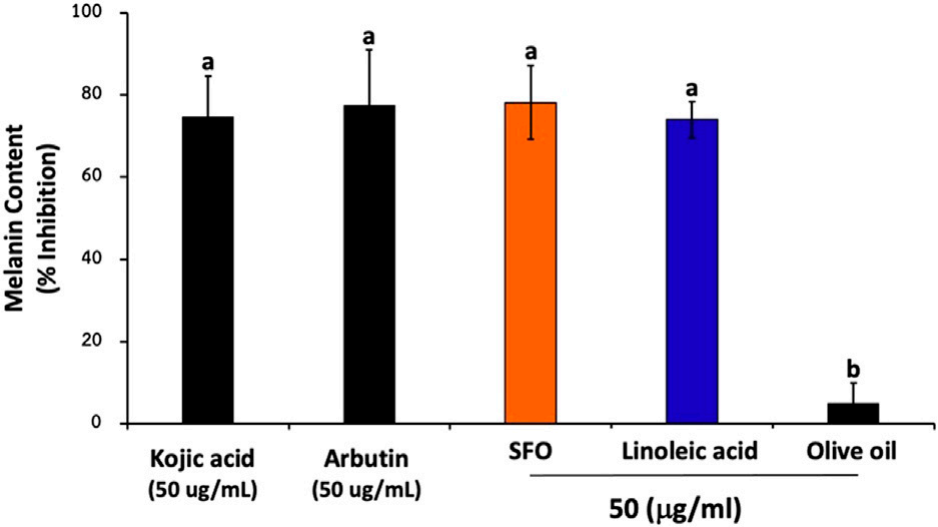
Effects of SFO on melanin content inhibition, B16F10 melanoma cells were treated with SFO, linoleic acid, olive oil, kojic acid or arbutin at a concentration of 50 μg/mL for 48 h at 37°C. After treatment, the cells were collected and washed with PBS. Then NaOH was added to the cells and incubated at 80°C for 30 min. The absorbance was measured at 405 nm using a microplate reader; (a and b denote significant differences among samples at *p* < 0.05).

As of today, in addition to anti-oxidant compounds, many cosmetic products contain antipigmenting active ingredients, such as kojic acid and arbutin, exhibiting skin whitening effects. To investigate whether SFO possesses antipigmenting properties, we assessed its ability to inhibit melanin content in B16F10 melanoma cells. As is shown in Figure 7, pretreating the cells with SFO led to reduced melanin content (78% inhibition). Furthermore, the level of melanin content inhibition achieved by SFO at the same concentration was comparable with that of well-known positive control agents, kojic acid and arbutin (75% and 77% inhibition, respectively). Conversely, olive oil at 50 µg/mL had little to no effect on melanin inhibition (5% inhibition). Meanwhile, linoleic acid, the major fatty acid present in SFO, exhibited similar inhibitory effects on melanin levels (74% inhibition). Our findings suggest that SFO, at a concentration of 50 µg/mL, can inhibit melanin content as effectively as well-known melanin pigment inhibitors. Therefore, SFO may be considered as a potent new antipigmenting agent.

## Discussion

This study provides scientific data on the SFO obtained from *Hermetia illucens* larvae. The physiochemical characteristics and lipid composition of the oil were determined and compared with other oils commonly used in cosmetic and supplement applications. Compared with another study, the acid value, saponification value and unsaponifiable matter of SFO revealed less than those reported in a related study (Mai et al., 2019). Briefly, the study reported the acid value of SFO at 11.8 mg KOH/g while its saponification value and unsaponifiable matter were 213 and 86 mg KOH/g, respectively. An increase or rise in acid value indicates the rancidification tendency of the oil. Taken together with the acid value, a measurement of potassium hydroxide (mg), required to neutralize the free fatty acid contained in unit mass (g) of chemical substance, the lower the acid value of oil, the fewer free fatty acids it contains making it less exposed to rancidification (Kardash and Tur’yan, 2005). Therefore, SFO in our study possessed a lower acid value and was less likely to generate the rancidity. A high saponification value suggested the presence of a fair amount of fatty acids but their acid values were low pointing to the fact that these fatty acids were not free but in an esterified form (Ekpo et al., 2009).

The fatty acid composition of SFO was compared with that of olive oil (*Olea europaea* L.), krill oil (*Euphausia superba*) and rice bran oil (*Oryza sativa*) from related studies (Orthoefer and Eastman, 2005; Ivanova et al., 2016). The data showed that olive oil contained a high amount of oleic acid (73.6%) followed by palmitic acid (14.11%) and linoleic acid (8.35%). Krill oil contained a high amount of palmitic acid (20.43%) followed by oleic acid (9.49%) and myristic acid (8.76%), while the highest fatty acid in rice bran oil was oleic acid (38.4%) followed by linoleic acid (34.4%) and palmitic acid (21.5%). In contrast, the most abundant component found in SFO was linoleic acid (C18:2 n-6 cis), which was consistent with related research (Barroso et al., 2017; Ewald et al., 2020). Linoleic acid has been shown to exhibit several pharmaceutical activities, especially regarding skin function (Huang et al., 2018). For example, it has been demonstrated to downregulate melanin synthesis, and clinical trials have reported the effectiveness of liposome containing 0.1% linoleic acid to treat melasma and lightening UVB-induced hyperpigmentation of the skin (Shigeta et al., 2004). Interestingly, linoleic acid was reported to positively correlate between amounts of linolenic acid and anti-oxidant activity (Saffaryazdi et al., 2020). Nevertheless, linoleic acid exhibited the ability to decrease in the epidermis with age causing skin sensitivity and roughness (Ahmad and Ahsan, 2020). Therefore, oils enriched with linoleic acid could be an interesting source for skin cosmetics. However, the lipid composition of BSFL significantly vary with their diets (Kim et al., 2020; Li et al., 2022). A higher percentage of omega-6 and 3 was obtained when the larvae were given the diet riches in polyunsaturated fatty acids from agricultural by-products (rape and flax cakes) (Hoc et al., 2021). Together with the work of Almeida et al. reported that the extraction technique significantly affects fatty acids profile of BSFL biomass (Almeida et al., 2022). Thus, it should be noted that consistency of batch-to-batch production should be highly considered to lessen variation of SFO lipid composition.

Several vegetable oils including rose hip, flax, hemp, thistle, safflower and camelina, have been chosen for cosmetic use due to their composition and high anti-oxidant activity. Anti-oxidants play a crucial role in preventing or reducing oxidative stress, leading to numerous health problems such as cancer, cardiovascular diseases, neurodegenerative disorders and skin aging. Natural anti-oxidants can inhibit or decrease the damage caused by oxidative stress through different mechanisms of action (Ionescu et al., 2015). Long term exposure to oxidative stress leads to redox imbalance, various biomolecules damage and abnormal gene expression, including those involved in aging pathways (Rungratanawanich et al., 2018). *In vitro* assays have demonstrated that SFO has higher radical scavenging, reducing power, and lipid peroxidation inhibition properties compared with rice bran oil, olive oil and lecithin. The anti-oxidant capacity of SFO was comparable with 5 mM Trolox (positive control) in the lipid peroxidation inhibition and slightly lower in the DPPH assays. However, the reducing capacity of SFO was much lesser than that of Trolox. Although linoleic acid, a major fatty acid in SFO did not exhibit strong anti-oxidant activity, SFO’s anti-oxidant activity may be due to other phenolic compounds, flavonoids, α-tocopherol and various fatty acids (Elagbar et al., 2016; El-Agbar et al., 2018). Studies have also shown that sterols and tocopherols/tocotrienols in insects' larvae oil may contribute to anti-oxidant capacity by acting as a chain-breaking activity (Mariod, 2013; dos Santos et al., 2021). Furthermore, PUFA, specifically alpha-linolenic acid and linoleic acid, have been found to be related to anti-oxidant activity (Alves et al., 2016). Phospholipids such as phosphatidylethanolamine and phosphatidylcholine can increase the ability of tocopherol to suppress lipid peroxidation (Bandarra et al., 1999; Judde et al., 2003; Takenaka et al., 2007). The polyunsaturated fatty acid moieties and amine groups in phospholipids can regenerate tocopheroxyl radicals to tocopherol (Henna Lu et al., 2011; Doert et al., 2012; Huang et al., 2020). These compounds have been shown to scavenge free radicals and prevent lipid oxidation, contributing to the overall anti-oxidant activity of SFO.

HA is a natural polysaccharide found in the extracellular matrix of vertebrate tissues, particularly soft connective tissues. It has a high molecular weight and consists of repeating disaccharide units of D-glucuronic acid and N-acetyl-D-glucosamine linked through (β-1,3 and β-1,4) glycosidic linkages. HA is naturally found in synovial fluid of joints, epidermis and dermis, where it serves as a lubricant for joints and a natural moisturizing factor that maintains skin moisture (Farage et al., 2008; Girish et al., 2009). HA degradation was attenuated when SFO was added to the HA-HAase system in hyaluronidase (HAase) inhibitory activity assay. HAase is a class of glycosidase that predominantly degrades HA, which is an extracellular matrix with a critical role in skin hydration and plumpness. Inhibition of this enzyme has received considerable attention in biological, pharmaceutical and cosmetic application (Girish et al., 2009). Since HAase is over activated and excessively breaks down HA, leading to the destruction of the proteoglycan network under chronic inflammation and/or oxidative stress (Hwang, 2010).

Linoleic acid, a representative major compound in SFO, was found to potentially be responsible for HAase inhibition activity, and other lipids such as oleanolic acid, eicosatrienoic acid and nervonic acid were reported for their HAase inhibitory activity (Girish et al., 2009). Suzuki et al. reported the inability of saturated fatty acid to inhibit the HA enzyme, whereas the cis-unsaturated fatty acids having double bonds are essential for inhibition (Suzuki et al., 2002). Molecular docking of the major components in SFO including linoleic acid, oleic acid and palmitic acid was carried out to investigate the binding interactions and molecular recognition of selected free fatty acids toward HAase. Interestingly, linoleic acid displayed the highest binding affinity to HAase when compared with other major fatty acids in SFO. The residue Y75 was found to be a crucial amino acid for binding HAase (Chao et al., 2007; Papaemmanouil et al., 2022). Reduction of HA degradation could lead to regulation of skin homeostasis, mitigates inflammatory and allergic states and diminish the aged appearance of skin (Hwang, 2010).

Based on our testing using MTT cell viability assay on HaCaT keratinocytes, primary human dermal fibroblasts, and peripheral blood mononuclear cells, we have found no cytotoxicity effects of SFO with the IC_50_ values exceeding 200 µg/mL. Other studies have also shown no cytotoxicity effects of oils derived from BSFL when tested on L-929 fibroblasts or using the *Artemia salina* model (Almeida et al., 2020; Franco et al., 2021; Riolo et al., 2023). Given these findings, we consider SFO to be safe for use in pharmaceutical and cosmetic products. To assess the *in vitro* biological effects of SFO in cell line models, we first tested its anti-oxidant activity by assessing its effects on UVB irradiation induced intracellular ROS in human dermal fibroblasts. The UVB-irradiation model in human dermal fibroblasts established in this study was modified from our described protocol (Mapoung et al., 2020). UV radiation can increase intracellular ROS levels and cause damage to the skin. Although the skin has a defense mechanism to prevent radical damage, this innate system can be overwhelmed, leading to aging and photo-aging. SFO’s strong anti-oxidant properties could potentially delay the skin’s aging process by counteracting the photo-aging process by reducing inflammatory responses (Jadoon et al., 2015; Xu et al., 2015). Although in this study we did not examine the anti-inflammation properties of SFO, previous studies have reported on the potential anti-inflammation properties of the major fatty acid of SFO, linoleic acid. Briefly, fatty acids, particularly, n-3 and n-6 essential fatty acids and conjugated linoleic acid (CLA), have been shown to modulate inflammation. Linoleic acid, an n-6 polyunsaturated fatty acid present in SFO, has been acknowledged for its potential anti-inflammatory properties (Saiki et al., 2017). Related studies have demonstrated that linoleic acid inhibits inflammatory responses in human THP-1 monocytes stimulated with LPS, leading to a decrease in the secretion of interleukin (IL)-6, IL-1β, and tumor necrosis factor-α (TNF-α) (Zhao et al., 2005). CLA isomers have also been found to possess anti-inflammatory effects by inhibiting ROS production and reducing the expression of inflammatory genes such as IL-6, IL-1beta and TNF-α in LPS-induced inflammation in bovine mammary epithelial cells (Dipasquale et al., 2018). Moreover, a Southern European nested case-control study (De Pablo et al., 2018) showed a significant inverse association (protective effects) between n-6 PUFA linoleic acid levels and pre-rheumatoid arthritis, indicating a lower risk of subsequent rheumatoid arthritis. Taken together, these findings suggest that the radical scavenging properties of SFO might be due to the presence of linoleic acid and that SFO may have anti-inflammatory effects potentially delaying the skin aging process. Nevertheless, the inhibitory effects of SFO and its major fatty acid (linoleic acid) on the cytokine production from skin cells should be confirmed.

In this study, we found that SFO and linoleic acid could promote the skin whitening effects not only through the removal of free radicals and the chelation of metal ions (antioxidant properties), but also through the depigmenting properties as evidence by the inhibition of melanin synthesis in B16F10 cells. Hyperpigmentation is a common skin condition that is particularly noticeable in individuals with darker skin tones and can be challenging to treat effectively. Numerous studies have been conducted by cosmetic scientists to identify skin-whitening agents that can address this issue, both through *in vivo* and *in vitro* research (Binic et al., 2013; Burger et al., 2016). One of the primary causes of hyperpigmentation is the overproduction of melanin by melanocytes, resulting in the formation of dark spots on the skin, particularly among older individuals and those with sun-exposed skin (Yodkeeree et al., 2018). To prevent or treat skin aging and hyperpigmentation, skincare products often utilize compounds that can protect the skin in various ways. These compounds may scavenge ROS, suppress ECM degradation enzymes, or inhibit melanogenesis. According to our results, SFO demonstrated a significant reduction in melanin synthesis by inhibiting melanin content in B16F10 melanoma cells, similar to well-known anti-pigmenting agents like kojic acid and arbutin. This suggests that SFO possesses potential whitening effects. Additionally, the major fatty acid found in SFO, linoleic acid, also exhibited inhibitory effects on melanin content. Linoleic acid has been previously associated with the modulation of melanogenesis and the inhibition of melanin synthesis in skin cells. Furthermore, other sources of linoleic acid, such as Chia seed extract (*Salvia hispanica*) and Passiflora edulis oil, have demonstrated the ability to inhibit melanin biosynthesis and reduce tyrosinase activity *in vitro* (Jorge et al., 2012; Diwakar et al., 2014). These findings suggested that compounds containing linoleic acid, like SFO, could be valuable in the prevention and treatment of hyperpigmentation and other signs of skin aging. Thus, our study supports the potential of SFO as a skin-whitening agent by inhibiting melanin synthesis. The presence of linoleic acid in SFO, along with previous research on mother sources of linoleic acid, indicates its involvement in the inhibition of melanin biogenesis. These findings contribute to the understanding of the mechanism of action behind the whitening effect of SFO and highlight the potential use of linoleic acid-containing compounds in combating hyperpigmentation and skin aging. Regarding the anti-skin aging properties, the strong antioxidant properties of the SFO could delay the aging process of the skin by counteracting the photo-aging process upon UV penetration. In addition, possible protective action of the SFO on the skin against ROS provoked and inhibition of HA degradation might serve as an anti-aging agent, mechanistically through reducing the inflammatory responses and oxidative stress, or by influencing specific survival signaling pathways, including MAPK and PI3K/Akt pathways. However, confirmation of the anti-skin aging effects of SFO and linoleic acid at the molecular level should be further investigated. The modulation of aged skin markers such as collagen type I/III ratio, fibroblast senescent markers (SA-β -gal activity, PTEN, and p53), and cell cycle regulator molecules (p16^INK4a^, p53) upon SFO treatment should be further identified.

## Conclusion

**FIGURE 8:**
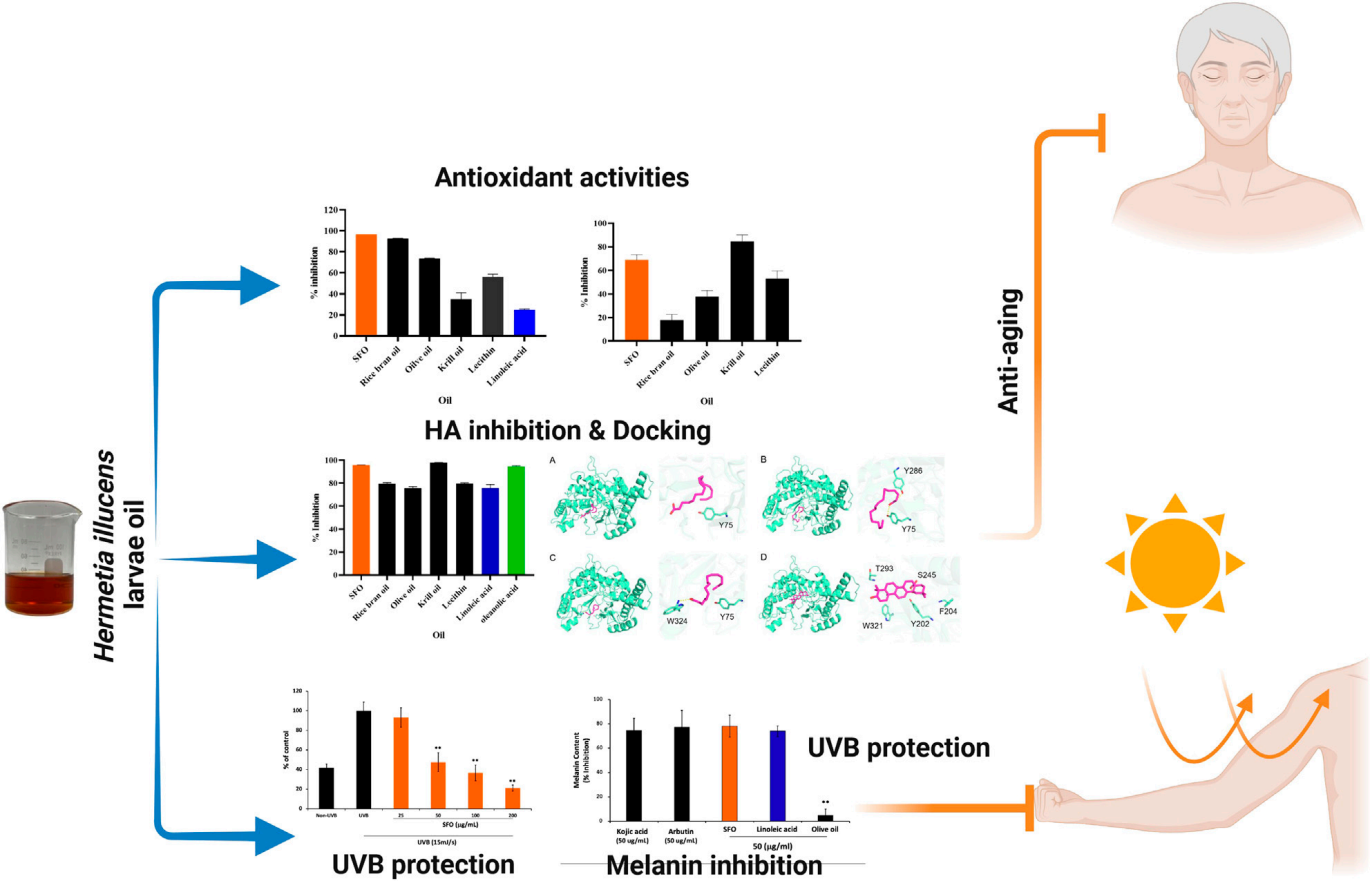
Pictorial summary illustrates the pharmacologic and cosmetic-related activities of black soldier fly oil. Figure was created with Biorender.com.

As of today, the search for novel compounds as sustainable active ingredients in cosmetic formulations has been an ongoing task in the industry. In our study, we have introduced and investigated the environmental-friendly and sustainable oil from *Hermetia illucens* larvae (SFO). SFO displayed its potential use in cosmetic applications as an alternative oil incorporated into the cosmetic formulation. We conducted a comprehensive analysis of the oil’s physicochemical characteristics, lipid composition and compared it with other commonly used oils in the cosmetic and supplement industry. Our findings suggest that SFO is a safe and effective ingredient for cosmetic products, with anti-oxidant properties both *in vitro* and *in situ*. In addition, we investigated the potential cosmetic properties of SFO and found it exhibited anti-hyaluronidase (anti-HAase) activity, which can be attributed to its linoleic acid and other oils content as determined by molecular docking. The SFO also demonstrated UVB protective effects in human dermal fibroblasts and anti-pigmenting effects in melanoma cell lines. As shown in a pictorial summary (Figure 8), our results suggest that SFO could constitute a promising ingredient in cosmetic products with implications for skin anti-aging, whitening, and UVB protection properties. Further studies on the *in vivo* effects of SFO and linoleic acid on anti-skin aging models should be conducted to confirm their efficacy before initiating testing on human subjects.

## Data Availability

The original contributions presented in the study are included in the article/Supplementary Material, further inquiries can be directed to the corresponding authors.
